# One particular *Anaplasma phagocytophilum* ecotype infects cattle in the Camargue, France

**DOI:** 10.1186/s13071-017-2305-3

**Published:** 2017-08-02

**Authors:** Thibaud Dugat, Agnès Leblond, Nicolas Keck, Anne-Claire Lagrée, Isabelle Desjardins, Aurélien Joulié, Sophie Pradier, Benoit Durand, Henri-Jean Boulouis, Nadia Haddad

**Affiliations:** 1 UMR BIPAR, Université Paris-Est, Agence nationale de sécurité sanitaire de l’alimentation, de l’environnement et du travail, Laboratoire de santé animale, Maisons-Alfort, France; 2 UR 0346 Épidémiologie Animale, INRA, Saint Genès Champanelle, France; 3 Equine Department, VetAgroSup, Marcy L’Etoile, France; 4 Laboratoire Départemental Vétérinaire de l’Hérault, Montpellier, France; 5 UMR BIPAR, Université Paris-Est, Ecole Nationale Vétérinaire d’Alfort, Maisons-Alfort, France; 6 IHAP, Université de Toulouse, INRA, ENVT, Toulouse, France; 7 Unité d’Épidémiologie, Université Paris-Est, Agence Nationale de Sécurité Sanitaire de l’alimentation, de l’environnement et du travail, Laboratoire de Santé Animale, Maisons-Alfort, France

**Keywords:** *Anaplasma phagocytophilum*, Camargue, Cattle, Horse, *Haemaphysalis punctata*, *Rhipicephalus pusillus*

## Abstract

**Background:**

*Anaplasma phagocytophilum* is a zoonotic tick-borne pathogen responsible for granulocytic anaplasmosis, a mild to a severe febrile disease that affects man and several animal species, including cows and horses. In Europe, *I. ricinus* is the only proven vector for this pathogen, but studies suggest that other tick genera and species could be involved in its transmission. Our objective was to assess the presence and genetic diversity of *A. phagocytophilum* in domestic animals and different tick species from the Camargue region, located in the south of France.

**Methods:**

A total of 140 ticks and blood samples from 998 cattle and 337 horses were collected in Camargue and tested for the presence of *A. phagocytophilum* DNA by *msp2* quantitative real-time PCR. Molecular typing with four markers was performed on positive samples.

**Results:**

*Anaplasma phagocytophilum* DNA was detected in 6/993 (0.6%) cows, 1/20 (5%) *Haemaphysalis punctata*, 1/57 (1.75%) *Rhipicephalus pusillus*, and was absent in horses (0%). All cattle *A. phagocytophilum* presented a profile identical to an *A. phagocytophilum* variant previously detected in *Dermacentor marginatus*, *Hyalomma marginatum*, and *Rhipicephalus* spp*.* in Camargue.

**Conclusions:**

Our results demonstrate that one particular *A. phagocytophilum* variant infects cattle in Camargue, where *I. ricinus* is supposed to be rare or even absent. *Dermacentor marginatus*, *Rhipicephalus* spp. and *Hyalomma* spp., and possibly other tick species could be involved in the transmission of this variant in this region.

**Electronic supplementary material:**

The online version of this article (doi:10.1186/s13071-017-2305-3) contains supplementary material, which is available to authorized users.

## Background

*Anaplasma phagocytophilum* is a tick-borne intragranulocytic alpha-proteobacterium that can infect many mammalian species. It is known to be the causative agent of tick-borne fever (TBF) in domestic ruminants, a disease with significant economic impact in Europe, and of equine granulocytic anaplasmosis (EGA) in horses in both the USA and Europe [1]. *Anaplasma phagocytophilum* also infects cats, dogs and humans and causes human granulocytic anaplasmosis (HGA), an emerging zoonotic disease in Asia, the USA, and Europe.

**Fig. 1:**
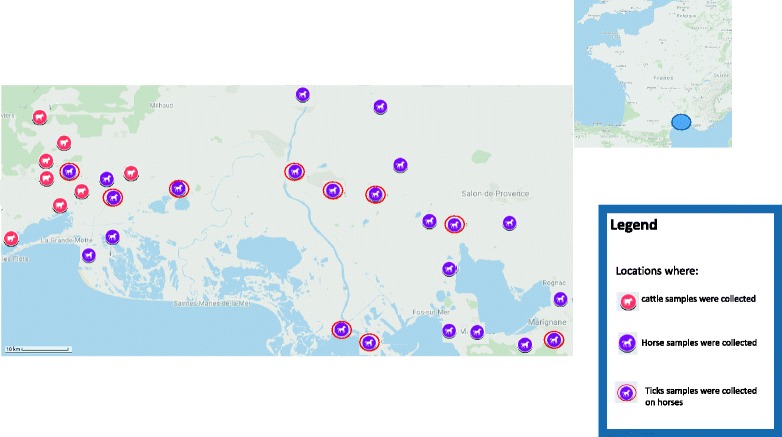
Location of the samples collected in this study. Each picture represents an animal host species: cattle, horse or ticks

*Anaplasma phagocytophilum* epidemiological cycles are complex and involve different ecotypes, vectors, and mammalian host species. To date, these epidemiological cycles are poorly understood, especially in Europe. Known arthropod vectors of *A. phagocytophilum* are ticks belonging to the genus *Ixodes*: *I. ricinus* in Europe, *I. scapularis* in eastern USA, *I. pacificus* and *I. spinipalpis* in the western USA, and *I. persulcatus* in Asia and Russia [1]. However, several studies suggest that other tick genera and species could be involved in *A. phagocytophilum* transmission, but their vector competence is yet to be proven [2]. For example, *I. trianguliceps* could play the role of an *A. phagocytophilum* vector in an independent epidemiological cycle in the UK and central Europe, involving different rodent species as reservoir hosts [3, 4]. In addition, it has been suggested that *Rhipicephalus* spp. could be involved in *A. phagocytophilum* transmission in the French Camargue region, a 1500 km^2^ marshland area in the south of France, and from where *I. ricinus* is supposed to be absent [5, 6]. Here, livestock is almost exclusively constituted of a local fighting bull breed, the Camargue breed, raised in extensive systems, which exposes them to arthropod bites. It is the same for the Camargue horses, which are also bred extensively. Even though up to 26% of horses have been found seropositive for *A. phagocytophilum* [7], live bacteria or bacterial DNA have never been detected in the animals. In addition, Camargue cattle have never been investigated for *A. phagocytophilum* infection. For these reasons, the presence/absence and the epidemiological cycle(s) of *A. phagocytophilum* in Camargue should be investigated.

Our objective was to assess the presence and genetic diversity of *A. phagocytophilum* in domestic animals and different tick species in Camargue. To achieve this, 140 ticks, and blood samples from 998 cattle (*Bos taurus*) and 337 horses (*Equus caballus*) were collected in Camargue and tested for the presence of *A. phagocytophilum* DNA by quantitative real-time PCR targeting the *msp2* gene. Positive samples were then molecularly typed *via* four sequences located within the genes *ankA*, *pleD*, *msp4* and *typA*, among those genes used for MLST by Chastagner et al. [5, 8].

## Methods

### Study area

Camargue is a 1500 km^2^ area in the south of France, from where *I. ricinus* has never been collected to date. Camargue is a region of marshlands where herds are almost exclusively constituted of a local fighting bull breed and a local horse breed raised in extensive conditions. Three French administrative departments extend into this area: Bouches-du-Rhône, Gard, and Hérault.

### Animal sampling

In 2015, blood samples from 337 horses (269 located in Bouches-du-Rhône, 47 in Gard, and 21 in Hérault) were collected. Blood from horses was preferentially collected near areas where ticks positive for *A. phagocytophilum* were detected in previous studies [5]. Ticks (engorged or not) feeding on horses was also collected during the same time. One-hundred and forty ticks were found on examined horses, 108 in Bouches-du-Rhône, 19 in Gard, and 13 in Hérault (Additional file 1: Table S1). Between 2013 and 2015, blood samples from 998 cattle (i.e. 10% of the 10,000 French Camargue cattle; 235 in Gard and 765 in Hérault), older than 24 months and belonging to the Camargue or Brave breeds, were collected (Fig. 1). These blood samples from cattle had been collected in a previous study unrelated to *A. phagocytophilum*, which explains why no tick sample was associated with these cattle samples. Moreover, collecting ticks on bovines in this region could be very hazardous as they are bred for fighting.

### Tick identification

Ticks were morphologically identified to the species level following Pérez-Eid’s recommendations [9].

### DNA extraction

For DNA extraction, the NucleoSpin® Blood QuickPure kit (Macherey-Nagel, Bethlehem, USA) (blood samples and engorged ticks), or the NucleoSpin® Tissue kit (Macherey-Nagel) (non-engorged ticks) were used according to manufacturer’s instructions. DNA extracts were then stored at -20 °C prior to testing.

### Quantitative real-time PCR

*Anaplasma phagocytophilum* DNA was detected by qPCR, targeting a 77 bp fragment of the *major surface protein 2* (*msp2*) gene as previously described by Courtney et al. [10]. To confirm the presence of *A. phagocytophilum* DNA, each *msp2* qPCR-positive sample was also tested for another gene specific to *A. phagocytophilum,* with qPCR targeting a fragment of the *ankA* and *groEL* genes, with the primers designed by Dugat et al. [11].

For each sample, qPCR targeting the bovine, equine and ticks *glyceraldehyde-3-phosphate dehydrogenase* (*gapdh*) gene was also performed to demonstrate the efficiency of DNA extraction and the absence of PCR inhibitors in the sample, using the primers designed [12, 13]. All samples had to be *gapdh* PCR-positive to be included in subsequent analyses.

For *ankA, groEL* and *gapdh*, qPCR assays were performed using the Maxima SYBR Green qPCR Master Mix (2×) Kit (Thermo Fisher Scientific, Villebon-sur-Yvette, France) in a 25 μl total reaction volume, with Master Mix at a 1× final concentration, 0.3 μM of each primer and 5 μl of purified DNA. Negative controls were included in each run. qPCR cycling was performed on the LightCycler480 Multiwell Plate 96 system (Roche, Basel, Switzerland) as follows: 95 °C for 10 min, then 40 cycles of 10 s at 95 °C, 30 s at 60 °C and 30 s at 72 °C. The signal emitted was detected at the end of each annealing-extension step. A threshold was automatically set, and the threshold cycle value (Ct) was consequently determined. All real-time PCR reactions were performed in duplicate.

### Genotyping

Samples positive for *A. phagocytophilum* were initially typed using the eight loci selected from a recently developed - MLST adapted for *A. phagocytophilum* [5, 8]. These loci were the following: *ankA*, *groESL*, *gyrA, msp4*, *pleD*, *polA*, *recG*, *typA*.

Sequencing results were analysed using Bioedit software version 7.2.5 (Ibis Biosciences, Carlsbad, USA). Each sequence was deposited in GenBank (KU859923–KU859946). Nucleotide sequences were then aligned to all the sequences available in GenBank, including those obtained by Chastagner et al. [5] (GenBank accession numbers: JX197073–JX197368) using the programme MEGA7 (Molecular Evolutionary Genetics Analysis Version 7.0.18) [14]. Gene sequences were aligned using ClustalW while applying the IUB matrix.

## Results

### Tick identification

A total of 140 ticks were collected from horses: 5 *Dermacentor reticulatus* (3.5%), 20 *Haemaphysalis punctata* (14.2%), 4 *Haemaphysalis* sp. (2.8%), 57 *Rhipicephalus pusillus* (40.4%), 19 *Rhipicephalus sanguineus* (13.5%), 1 *Rhipicephalus turanicus* (0.7%), and 34 *Rhipicephalus* sp. (24.2%). None of the 140 collected ticks belonged to the genus *Ixodes*.

### Detection of *A. phagocytophilum* DNA

For domestic animals, 993/998 (99.5%) cow samples and 269/337 (79.8%) horse samples gave positive *gapdh* PCR results and were included in subsequent analyses. 6/993 (0.6%, 95% CI: 0.2–1.3%) cow samples were *msp2* PCR-positive. These six positive cows belonged to the same herd, located in Hérault, and all cows had been born in this herd. No *msp2* amplification was observed in any of the 269 horses tested (0%, 95% CI: 0–1.4%) (Table 1, Additional file 1: Table S1). Statistically, cattle were not more frequently infected by *A. phagocytophilum* than horses (Fisher’s exact test, *P* = 0.35).

**Table 1:** Prevalence of *A. phagocytophilum* infection for each animal species

Species	No. of *msp2*-positive/total no.	(%)	95% CI
Cow (*B. taurus*)	6/993	0.6	0.2–1.3
Horse (*E. cabalus*)	0/269	0	0–1.4
*Dermacentor reticulatus*	0/5	0	
*Haemaphysalis punctata*	1/20	5	0–36
*Haemaphysalis* sp.	0/4	0	
*Rhipicephalus pusillus*	1/57	1.75	0–5.17
*Rhipicephalus sanguineus*	0/19	0	
*Rhipicephalus turanicus*	0/1	0	
*Rhipicephalus* sp.	0/34	0	

For the tick samples, 137/140 (97%) were *gapdh* PCR-positive and were included in subsequent analyses. Only 2/137 (1.5%, 95% CI: 0.2–5.2%) were *msp2* PCR-positive: one *R. pusillus* male and one *H. punctata* male (Table 1, Additional file 1: Table S1). These two ticks were collected from two different horse farms next to the town of Arles (Bouches-du-Rhône).

The eight *msp2* PCR-positive samples were also positive for *ankA* and *groEL* qPCRs.

### Genotyping

In the positive *H. punctata* and *R. pusillus*, DNA quantity was too low to enable complete genotyping. However, genotyping could be performed on the six positive cow samples. All eight genes included in this technique gave positive PCR results, but positive sequencing results were generated only for four loci: *ankA*, *pleD*, *msp4*, and *typA*. The sequence quality of *groESL*, *gyrA*, *recG* and *polA* loci was too poor to be analysed properly. Subsequently, too little sample DNA remained to perform a second round of genotyping. All six cow samples presented 100% identity at these four loci. The sequences of these genes were then aligned to all the sequences available on GenBank, including those obtained by Chastagner et al. [5]. For all four loci, the six positive cow samples shared 100% identity with the single *A. phagocytophilum* genotype identified in 40 *Rhipicephalus* spp., *D. marginatus* and *H. marginatum* [5]. However, when comparing the *msp4* sequences, the Camargue genotype was located in a different cluster than *A. phagocytophilum* variants from cattle living in several other French areas [14]. Moreover, a significant part of the available sequences of *A. phagocytophilum* from humans in different areas in the USA (HGE1, HZ, NY18 and Webster) were also located in this cluster, as well as one sequence from a horse living in the USA (Additional file 2: Table S2). Apart one sequence from a red deer in Slovenia and one sequence from a donkey in Italy, they were the only non-Camargue samples to belong to this cluster.

## Discussion

*Anaplasma phagocytophilum* can infect many mammalian species worldwide and is known to be the causative agent of TBF and EGA, two diseases with high economic impact in Europe [1]. On this continent, *I. ricinus* is the main vector, and to date the only proven vector, of *A. phagocytophilum*. In the present study, we investigated for the first time the presence and genetic diversity of *A. phagocytophilum* both in ticks and domestic animals in Camargue, a 1500 km^2^ area in the south of France from where *I. ricinus* is supposed to be absent, due to unfavourable ecosystem conditions for this species.

To our knowledge, this study is the first large-scale screening of *A. phagocytophilum* in cattle from a particular French region and is the first to report *A. phagocytophilum* DNA in cows from Camargue. Six on 998 cows, which all belonged to the same herd, were found positive for *A. phagocytophilum*. Our results are consistent with those obtained by Torina et al. [15] in Sicily (5/374 cows, 1.3%, 95% CI: 0.4–3.1%), a region in which *I. ricinus* is rare, whereas *Dermacentor marginatus*, *H. marginatum* and *Rhipicephalus* spp. are commonly collected. Our results demonstrate that *A. phagocytophilum* infects cattle in Camargue.

However, this region is considered a *I. ricinus*-free area: indeed, no *I. ricinus* were collected in our study or during several studies conducted between 2007 and 2010 [16]. Moreover, *A. phagocytophilum* DNA has already been detected in *R. bursa*, *R. sanguineus*, *R. turanicus*, *R. pusillus*, *D. marginatus* and *H. marginatum* in Camargue [5]. Taken together, these results indicate *A. phagocytophilum* is most likely transmitted by the vector(s) other than *I. ricinus* in this region. Thus we investigated the presence of *A. phagocytophilum* in different tick genera and species collected in Camargue. At the level of our sampling, only two ticks were qPCR-positive: one *R. pusillus* male and one *H. punctata* male. This is the first report of *A. phagocytophilum* in *R. pusillus*, whereas *H. punctata* has recently been suspected to be a vector of *A. phagocytophilum* in Spain [17]. This species could then also potentially transmit *A. phagocytophilum* to cows in Camargue. However, the high number of *A. phagocytophilum*-infected tick species found in Camargue from previous studies raises questions about their vector competence. Many of these ticks could have acquired *A. phagocytophilum* (or only its DNA) by passively feeding on an infected animal, without then being able to transmit the pathogen. Unfortunately, due to the low quantity of tick sample DNA, we were not able to determine the genotype of *A. phagocytophilum* present in these ticks and compare it to the genotype present in the cows typed in our study. However, it is noteworthy that the profile of the six *A. phagocytophilum* cow samples for the four tested genes (*ankA*, *pleD*, *msp4* and *typA*) was identical to that of *A. phagocytophilum* from 40 *Rhipicephalus* spp., *D. marginatus* and *H. marginatum* sampled in Camargue during a previous study [5]. This profile was shared by ticks that had been collected throughout the three French administrative departments of Camargue, which covers 1500 km^2^. In addition, this genotype has never been detected in ticks and animals from other regions in France. These observations strongly suggest that only one variant, which is transmitted by one or several tick species, infects cows in Camargue in an epidemiological cycle independent from *I. ricinus*. This “specialisation” could have led to decreased *A. phagocytophilum* diversity in Camargue, resulting in the circulation of this single variant.

Interestingly, the *msp4* cluster to which this variant belongs also includes sequences originating from humans in the USA. It is particularly remarkable to notice that this a priori result is in complete accordance with that previously observed using MLVA, with identical profiles between the samples from Camargue and the human Webster profile, the only American human sample that could be tested by MLVA [6]. The sequence identity, for both markers, of samples originating from regions as far apart from one another (Camargue and the USA) could be due to homoplasy.

None of the 269 studied horses was infected by *A. phagocytophilum*, whereas in prior studies occurring between 2001 and 2010, 8.6 to 26% of examined horses were seropositive [7, 16, 18]. In this context, the absence of any positive PCR result in horses in our study was a priori surprising as 42 out of the 337 horses tested in the present study displayed clinical signs compatible with equine granulocytic anaplasmosis. This could be explained by the fact that most of the diseased horses received imidocarb. This treatment was administered because *Theileria equi* infection is the main cause of fever in horses in this area. Imidocarb is also commonly used for the treatment of cattle anaplasmosis. Thus, the use of this babesicid molecule could most likely explain the negative PCR results.

Finally, the reservoir host(s) of the Camargue variant must be identified. This unique and stable variant suggests that it could be adapted to a restricted number of reservoir host(s) and/or vector(s). This variant belongs to the *ankA* cluster I, which mostly contains variants obtained from humans, dogs, cats, and horses, and several variants obtained from domestic and wild ruminants [5, 8]. *Haemaphysalis punctata* females are known to have a trophic preference for both wild and domestic ruminants, and *R. pusillus* for rabbits [9]. For these reasons, wild ruminants and rabbits should be investigated as potential *A. phagocytophilum* reservoirs in Camargue, even if there is some doubt about the vector competence of *H. punctate* and *R. pusillus*. Furthermore, it also remains to be demonstrated that the same unique variant is also present in these ticks.

Due to these observations, prevalence studies should be continued in both ticks and domestic animals throughout the coming years in order to determinethe *A. phagocytophilum* vector competence of the tick species present in Camargue, the reservoir host of *A. phagocytophilum* in Camargue, the level of infection in cattle and horses and the clinical impact of disease in these species, and the zoonotic potential of this variant.

## Conclusion

In conclusion, our results strongly suggest that one particular *A. phagocytophilum* variant infects cows in Camargue, an area where *I. ricinus* is supposed to be rare or even absent. A variant that presented the same profile based on our four markers was already identified in *Rhipicephalus* spp., *D. marginatus*, *H. marginatum*, *H. punctata* and *R. pusillus* by Chastagner et al. [5]. These ticks could be involved in *A. phagocytophilum* transmission in this particular region, but additional studies are needed before confirming this theory. The vertebrate and invertebrate actors of this epidemiological cycle must now be confirmed or identified to develop appropriate surveillance measures. Finally, the zoonotic potential of this variant should also be investigated.

## Additional files

Additional file 1: Table S1. Characteristics and qPCR results for the samples used in this study. (XLSX 67 kb)

Additional file 2: Table S2. Phylogenetic tree of *msp4* sequences of *A. phagocytophilum* available in the GenBank database. (PDF 26 kb)

## Abbreviations

EGA: Equine granulocytic anaplasmosis
*gapdh*: Glyceraldehyde-3-phosphate dehydrogenase
HGA: Human granulocytic anaplasmosis
MLST: Multilocus sequence typing
msp2: Major surface protein 2
qPCR: Quantitative real-time PCR
TBF: Tick-borne fever

## References

[1] Dugat T, Lagrée A-C, Maillard R, Boulouis H-J, Haddad N. Opening the black box of *Anaplasma phagocytophilum* diversity: current situation and future perspectives. Front Cell Infect Microbiol. 2015;61 10.3389/fcimb.2015.00061 PMC4536383 26322277

[2] Stuen S, Granquist EG, Silaghi C. *Anaplasma phagocytophilum -* a widespread multi-host pathogen with highly adaptive strategies. Front Cell Infect Microbiol. 2013;3(31) 10.3389/fcimb.2013.00031 PMC3717505 23885337

[3] Blaňarová L, Stanko M, Carpi G, Miklisová D, Víchová B, Mošanský L (2014). Distinct *Anaplasma phagocytophilum* genotypes associated with *Ixodes trianguliceps* ticks and rodents in Central Europe. Ticks Tick-Borne Dis.

[4] Bown KJ, Lambin X, Ogden NH, Begon M, Telford G, Woldehiwet Z (2009). Delineating *Anaplasma phagocytophilum* ecotypes in coexisting, discrete enzootic cycles. Emerg Infect Dis.

[5] Chastagner A, Bailly X, Leblond A, Pradier S, Vourc’h G (2013). Single genotype of *Anaplasma phagocytophilum* identified from ticks, Camargue. France Emerg Infect Dis.

[6] Dugat T, Chastagner A, Lagrée A-C, Petit E, Durand B, Thierry S (2014). A new multiple-locus variable-number tandem repeat analysis reveals different clusters for *Anaplasma phagocytophilum* circulating in domestic and wild ruminants. Parasit Vectors.

[7] Leblond A, Pradier S, Pitel P, Fortier G, Boireau P, Chadoeuf J (2005). Enquête épidémiologique sur l’anaplasmose équine (*Anaplasma phagocytophilum*) dans le sud de la France. Rev Sci Tech Off Int Epizoot.

[8] Chastagner A, Dugat T, Vourc HG, Verheyden H, Legrand L, Bachy V (2014). Multilocus sequence analysis of *Anaplasma phagocytophilum* reveals three distinct lineages with different host ranges in clinically ill French cattle. Vet Res.

[9] Pérez-Eid C (2007). Les tiques, identification, biologie, importance médicale et vétérinaire.

[10] Courtney JW, Kostelnik LM, Zeidner NS, Massung RF (2004). Multiplex real-time PCR for detection of *Anaplasma phagocytophilum* and *Borrelia burgdorferi*. J Clin Microbiol.

[11] Dugat T, Loux V, Marthey S, Moroldo M, Lagrée A-C, Boulouis H-J (2014). Comparative genomics of first available bovine *Anaplasma phagocytophilum* genome obtained with targeted sequence capture. BMC Genomics.

[12] Bougarn S, Cunha P, Gilbert FB, Meurens F, Rainard P (2011). Technical note: validation of candidate reference genes for normalization of quantitative PCR in bovine mammary epithelial cells responding to inflammatory stimuli. J Dairy Sci.

[13] Kayis SA, Atli MO, Kurar E, Bozkaya F, Semacan A, Aslan S (2011). Rating of putative housekeeping genes for quantitative gene expression analysis in cyclic and early pregnant equine endometrium. Anim Reprod Sci.

[14] Tamura K, Stecher G, Peterson D, Filipski A, Kumar S. MEGA6: Molecular Evolutionary Genetics Analysis version 6.0. Mol Biol Evol. 2013;30:2725–9. 10.1093/molbev/mst197 PMC3840312 24132122

[15] Torina A, Alongi A, Naranjo V, Estrada-Peña A, Vicente J, Scimeca S (2008). Prevalence and genotypes of *Anaplasma* species and habitat suitability for ticks in a Mediterranean ecosystem. Appl Environ Microbiol.

[16] Leblond A, Chastagner A, Pradier S, Bailly X, Masseglia S, Vourc’h G. La prévalence de l’anaplamose dans le sud de la France. Bull Épidémiologique Santé Anim Aliment. 2012:30–1.

[17] Palomar AM, Portillo A, Santibáñez P, Mazuelas D, Roncero L, García-Álvarez L (2015). Detection of tick-borne *Anaplasma bovis*, *Anaplasma phagocytophilum* and *Anaplasma centrale* in Spain. Med Vet Entomol.

[18] Tilliette B (2008). Anaplasmose granulocytaire équine: enquête sero-épidémiologique dans le sud est de la France en 2007 [Veterinary thesis].

