# Supplementary figures and images for: One particular Anaplasma phagocytophilum ecotype infects cattle in the Camargue, France
Source: Parasit Vectors. 2017 Aug 2;10:371. doi: 10.1186/s13071-017-2305-3 (PMC5540577; doi:10.1186/s13071-017-2305-3)

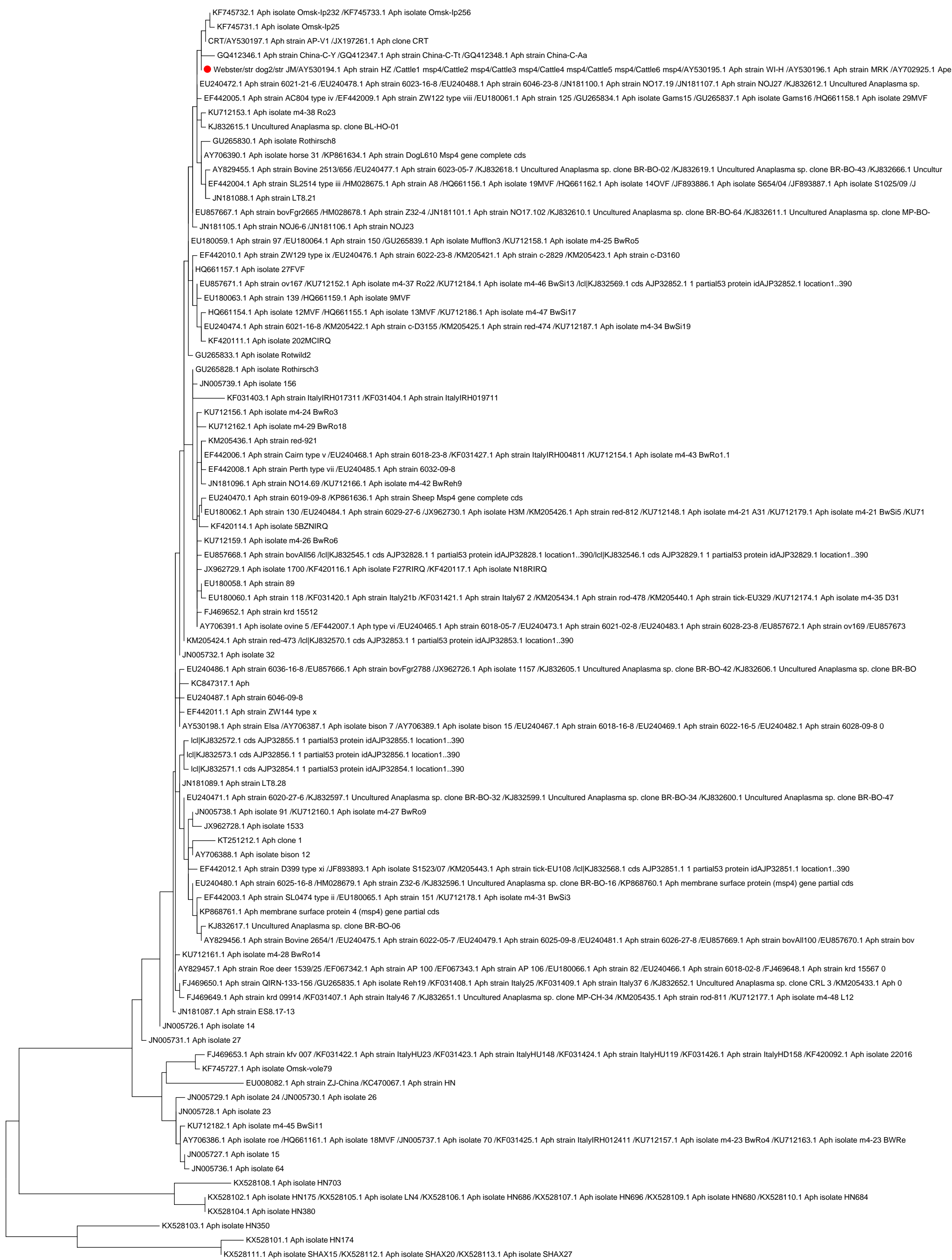

Supplement: Supplementary file 2 — Phylogenetic tree of msp4 sequences of A. phagocytophilum available in the GenBank database. (PDF 26 kb) [file 13071_2017_2305_MOESM2_ESM.pdf]
